# The value of brainstem auditory evoked potentials in the diagnostic assessment of dysphagia after craniocerebral injury

**DOI:** 10.3389/fneur.2026.1838871

**Published:** 2026-10-05

**Authors:** Zhen-Hai Fei, Xing-Ming Zhong, Yong Cai, Li-Ying Dong

**Affiliations:** 1 Neurosurgery, First Affiliated Hospital of Huzhou Normal University, Huzhou, China; 2 Neurosurgery, The First People’s Hospital of Huzhou, Huzhou, China

**Keywords:** brainstem auditory evoked potential, craniocerebral injury, dysphagia, neurology, neurorehabilitation

## Abstract

**Purpose:**

The aim of this study was to explore the value of brainstem auditory evoked potentials in the diagnostic assessment of dysphagia after craniocerebral injury.

**Methods:**

To investigate the role of brainstem auditory evoked potentials (BAEPs) in dysphagia diagnosis, we prospectively analyzed BAEP findings and gold-standard diagnoses in 106 patients with head injury. Inclusion criteria: ① Age 18–70 years old; ② Diagnosed by Chinese Expert Consensus on Evaluation and Treatment of Dysphagia. Exclusion criteria: ① Patients with a Glasgow coma scale score ≤8 and patients who cannot be managed with intervention; ② Previous comorbidities that may affect swallowing function.

**Results:**

A total of 65 males and 41 females patients were enrolled. The positive rate of BAEP in moderate to severe dysphagia was 86.96%, which was significantly higher than that in the mild dysphagia group (45.95%; *p* < 0.01). 60 patients were BAEP-positive and moderate-to-severe dysphagia patients, and considered neurogenic dysphagia, 46 patients were BAEP-negative, and considered non-neurophagia. The AUC of BAEP diagnosis of moderate to severe dysphagia was 0.705.

**Conclusion:**

BAEP can be used as an accurate diagnostic method for nerve injury, it is helpful to identify whether it is a neurological dysphagia.

## Introduction

With the development of emergency medicine and critical care medicine, the survival rate of patients with craniocerebral injuries has been substantially improved, but along with it, the disability rate of patients is also increasing year by year (1). According to statistics, nearly 30–73% of patients with craniocerebral injuries will suffer from neurogenic swallowing dysfunction, which will lead to serious consequences such as aspiration pneumonia, dehydration, hypoproteinemia, and so on, of which aspiration pneumonia is even more related to the prognosis of the patients, and may even lead to an increase in the rate of death (2, 3).

Post-traumatic dysphagia can be categorized into structural dysphagia and neurological dysphagia (ND) according to the pathogenesis (4). The main causes of the former include inflammation, injury, or tumor of the swallowing passage or adjacent organs, which usually results in better functional recovery after symptomatic treatment. In contrast, neurological dysphagia tends to have a poorer prognosis and currently lacks effective rehabilitative treatments, which should be targeted as early as possible to minimize functional loss (5).

When dealing with patients with post-traumatic dysphagia, it is necessary to distinguish between neurologic and structural dysphagia. Currently, the diagnosis of neurogenic dysphagia is mostly based on clinical symptoms, and relevant auxiliary tests such as the puddle-drinking test, GUSS Scores can only provide indirect evidence of neurologic injury, and these tests cannot effectively differentiate between neurologic and non-neurologic injury-induced dysphagia. Therefore, the difficulty in diagnosing dysphagia often delays the treatment of aspiration pneumonia, and the search for an early and effective adjunctive test for the early diagnosis of dysphagia is urgent and necessary (4).

Brainstem auditory evoked potential (BAEP), also known as auditory brainstem response (ABR), is a series of neurogenic electrical activities induced by acoustic stimulation with a latency of less than 10 milliseconds. Since the amplitude of neuronal activity induced by a single stimulation is only 1/100 of the amplitude of normal brain wave, a clear waveform can be obtained by computer superposition (6). BAEP has become a commonly used neurophysiological detection technique in clinical practice, and the technique is extremely sensitive to neurophysiology, and abnormal changes can be seen even when there is a mild brainstem injury presenting with no obvious clinical signs and symptoms (7, 8). In previous studies, BAEP has shown good diagnostic value in the detection of abnormal swallowing function in groups such as postoperative patients with hemifacial spasm (HFS) and patients with spinocerebellar ataxia (9, 10). However, the early diagnostic value of BAEP for swallowing disorders in patients with craniocerebral injuries has not been confirmed by effective studies.

## Methods

### Study participants

Patients with dysphagia after craniocerebral injury who were admitted to the Department of Neurosurgery were selected for this study. Inclusion criteria: Inclusion criteria were as follows: patients aged between 18 and 70 years, of either gender, with traumatic brain injury confirmed by cranial computed tomography (CT) or magnetic resonance imaging (MRI), and diagnosed with dysphagia according to the Chinese Expert Consensus on the Assessment and Treatment of Dysphagia (11). Additionally, patients were required to have a Glasgow Coma Scale (GCS) score greater than 9, stable vital signs, and the ability to cooperate with all examinations. Written informed consent was obtained from each patient or their legal representative after a full explanation of the study protocol.

Exclusion criteria included: (a) previous or current otitis media, hearing impairment, history of ear surgery, or other diseases of the auditory pathway that could potentially affect the results of BAEP testing; (b) severe psychiatric disorders or cognitive impairment, or current use of high dose sedatives, muscle relaxants, or other medications that might significantly interfere with BAEP recordings and swallowing reflex assessments; (c) concurrent severe cardiac, pulmonary, hepatic, or renal insufficiency, or uncorrected internal milieu disturbances such as severe infection or electrolyte imbalance; (d) history of epilepsy, or a history of alcohol or psychoactive substance abuse/dependence; and (e) pregnancy, lactation, or concurrent participation in other interventional clinical trials. This study has been registered in the Chinese Clinical Data Center with the registration number of ChiCTR2500104698.

### Evaluation of dysphagia and related ancillary examination

GUSS Scores was a clinical test of swallowing liquids, pastes, and solids, with the next food being swallowed only after completion of the previous one, thus reducing the risk of patient aspiration and improving safety, which can be used to screen patients at high risk for swallowing dysfunction in craniocerebral injury (12, 13). The scale determines dysphagia based on a score of 0–19, where 0–9, 10–14, and 15–19 correspond to severe, moderate, and mild dysphagia in that order, and 20 is the presence of no dysphagia. The NIHSS score was used to evaluate the severity of neurological deficits (14). The scale includes 11 items and 15 items, including consciousness, gaze, facial paralysis, upper limb muscle strength, lower limb muscle strength, ataxia, aphasia, sensory, visual field, neglect syndrome, and distal limb function, with a total score of 0 ~ 42 points, the higher the score, the more serious the neurological deficit.

### BAEP test

All patients underwent bedside BAEP at a random time early in admission (≤24 h) and were performed by formally trained, discipline-compliant technicians. Neuroimaging studies (CT and MRI) were conducted at admission and at selected time points during the clinical course, as dictated by clinical necessity; the localization of lesions was determined based on a comprehensive evaluation of all available imaging data. Although the BAEP recordings and imaging examinations were not strictly synchronized, both were carried out as early as possible once the patients’ clinical condition had stabilized and appropriate testing conditions were met. The instrument used was a BAEP module of a Medelec Synergy neuromyography (OXFORD, UK). Keep the room quiet and avoid external interference as much as possible. Before the test, the subject is instructed to relax and rest quietly, and ask him to put his hands at his sides, wipe with alcohol, and let them dry. The needle electrode is placed in the median position of the cranial roof, the reference electrode is placed on the donal side of the mastoid, and the contralateral side of the mastoid process is grounded. The stimulus is a short sound with a frequency of 10 beats/s and an intensity of 60 dBnHL. The filtering parameters were 30–3,000 Hz, and the sampling time was 10 ms. The amplitude is directly read out by the tester, and the latency, inter-wave interval and waveform of waves I., III, V. of the BAEP examination chart are recorded. Each check is repeated at least 2 times and averaged to ensure repeatability of waves I, III, V (7).

### Statistical analysis

All data were collected using Excel and accurately entered into SPSS 26.0 software for statistical analysis. Continuous data (age, GCS Scores, GUSS Scores, NIHSS Scores, etc.) were expressed as mean ± SD; whereas categorical data like gender, site of injury, cause of injury, etc. were expressed as frequency and percentage. Chi-square test was used to analyze the relationship between the degree of dysphagia and the abnormal rate of BAEP test, and the ROC curve of BAEP in diagnosing the degree of dysphagia was plotted.

### Ethical approval

This study has been reviewed and approved by the hospital’s ethical review committee (approval No.2023KYLL052), and all patients participating in this study signed an informed consent form.

## Results

### General information and grouping

From January 2018 to December 2020, a total of 106 patients who met the diagnosis of craniocerebral injury were included in this study, including 65 males and 41 females, with an average age of (42.980 ± 13.01) years, including 38 traffic accident injuries, 34 fall injuries from heights, 29 self-inflicted falls, 5 falling objects. Some patients had impaired consciousness on admission, but had recovered to the point where they were able to cooperate in completing the assessment at the time of diagnosis of dysphagia. Detailed basic information is shown in Table 1.

**Table 1:** Inclusion of demographic and clinical characteristics of included patients.

Variables		Total (*n* = 106)
Age (years, *x* ± *s*)		42.980 ± 13.01
Gender (%)	Male	65 (61.32%)
	Female	41 (38.68%)
Causes of injury (%)	Traffic accidents	38 (35.85%)
	Falls from heights	34 (32.06%)
	Self-inflicted falls	29 (27.36%)
	Falling objects	5 (4.72%)
Sites of injury (%)	Frontal	13 (12.26%)
	Temporal	14 (13.21%)
	Parietal	15 (14.15%)
	Occipital	12 (11.32%)
	Thalamus	13 (12.26%)
	Cerebellum	16 (15.09%)
	Basal ganglia	10 (9.43%)
	2 or more sites	13 (12.26%)
GCS (points, *x* ± *s*)		10.80 ± 1.934
GUSS (points, *x* ± *s*)		13.10 ± 2.828
NIHSS (points, *x* ± *s*)		15.43 ± 4.045

Table 1 presents the detailed demographic and clinical characteristics of the 106 patients included in the study. The table includes information on age, gender, causes of injury, sites of injury, Glasgow Coma Scale (GCS) score, Gugging Swallowing Screen (GUSS) score, and National Institutes of Health Stroke Scale (NIHSS) score.

### Evaluation of swallowing function and neurologic deficits

All 106 patients were evaluated for swallowing function and nerve damage within 1 week of admission. According to the results of GUSS assessment of dysphagia: the number of cases of mild, moderate, and severe dysphagia were 37 (34.91%), 57 (53.77%), and 12 (11.32%), respectively. Secondly, according to the NIHSS assessment: the number of cases of moderate, moderately severe, and severe neurological damage were 64 (60.38%), 26 (24.53%), and 16 (15.10%), respectively.

### BAEP test

There were 77 cases with abnormal BAEP test results (72.64% positive rate). I-wave latency and wave amplitude were abnormal in 77 abnormal cases, of which 20 (25.97%) had abnormal III-wave latency and wave amplitude and 16 (19.48%) had abnormal V-wave latency and wave amplitude.

According to the degree of dysphagia, the positive rate of BAEP detection and the distribution of abnormal waveforms in the three groups were analyzed. The BAEP positivity rate was the highest in the moderate dysphagia group, which was 92.98%. There was a statistically significant difference in the positive rate of BAEP between the three groups (*p* < 0.01), Table 2.

**Table 2:** The results of BAEP detection and GUSS grade were analyzed in 106 patients.

BAEP test	GUSS Severity					
	Mild (*n* = 37)	Moderate (*n* = 57)	Severe (*n* = 12)	Total (*n* = 106)	*χ* ^2^	*p*
BAEP positive	17	53	7	77 (72.64%)	26.371	<0.01
I-wave positive	17	53	7	77 (72.64%)	26.371	<0.01
III-wave positive	2	14	4	20 (18.87%)	7.228	0.027
V-wave positive	1	11	4	16 (15.09%)	8.334	0.015

Table 2 analyzes the relationship between brainstem auditory evoked potential (BAEP) test results and the severity of dysphagia as assessed by the Gugging Swallowing Screen (GUSS) in 106 patients. The table categorizes patients into groups based on the severity of dysphagia (mild, moderate, severe) and presents the BAEP positivity rates along with abnormalities in wave I, III, and V. Additionally, the table provides chi-square test results and *p*-values to assess the statistical significance of the differences in BAEP positivity rates across different dysphagia severity groups.

According to the above results, a four-panel table is plotted, and the ROC curve is plotted. The sensitivity was 86.96% (60/69), the specificity was 54.05%, and the area under the ROC curve was 0.705, Figure 1.

**Figure 1:**
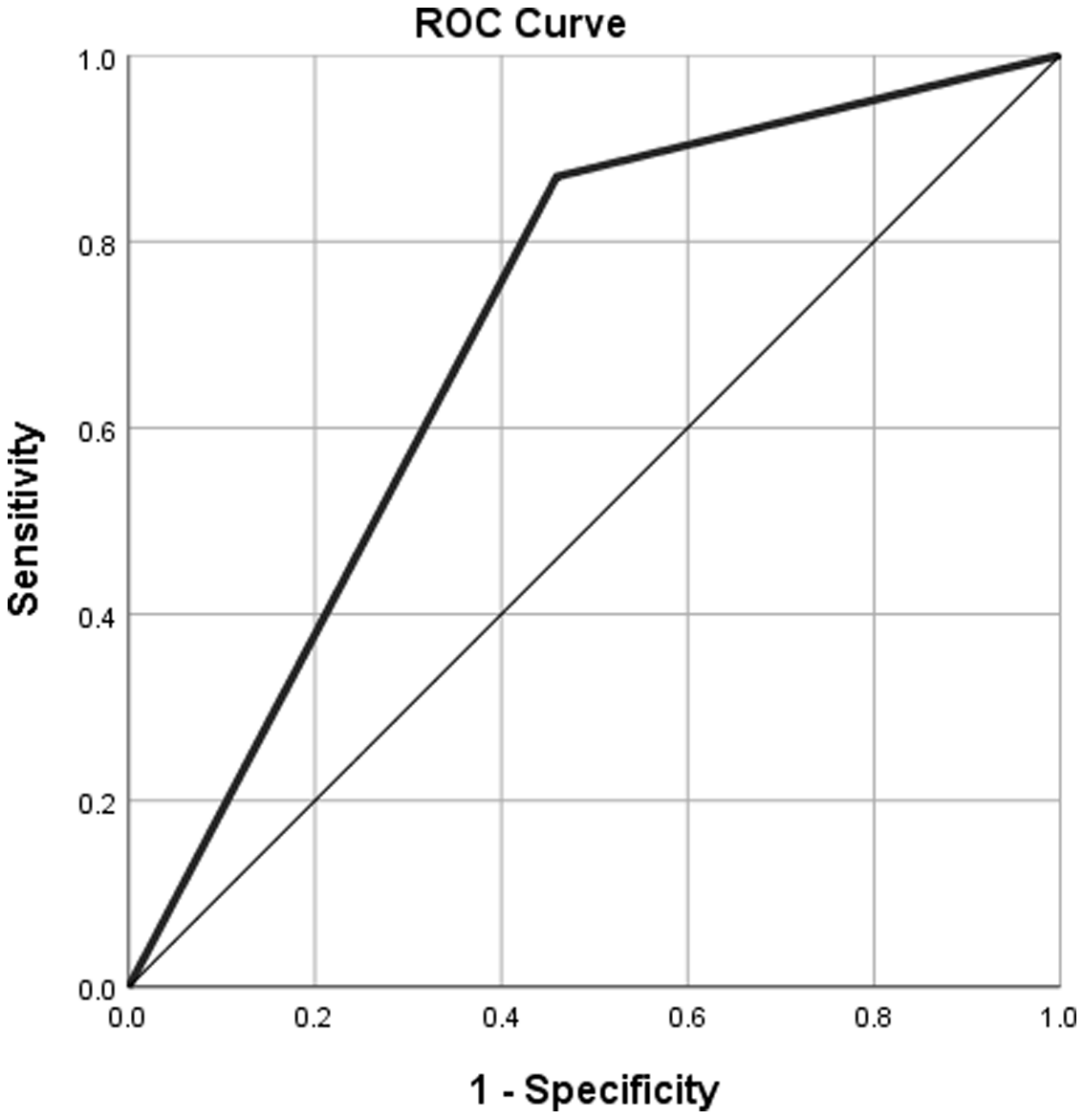
ROC curve of BAEP in diagnosing the degree of dysphagia.

Figure 1 shows the receiver operating characteristic (ROC) curve of brainstem auditory evoked potentials (BAEP) in diagnosing the degree of dysphagia. The sensitivity was 86.96% (60/69), the specificity was 54.05% (20/37), and the area under the ROC curve (AUC) was 0.705. This figure illustrates the diagnostic accuracy of BAEP in identifying moderate to severe dysphagia.

## Discussion

The innervation of swallowing function is mainly composed of three parts: the swallowing center in the cerebral cortex, the nerve conduction centers in the brainstem, and the peripheral nerves (glossopharyngeal nerve, hypoglossal nerve, vagus nerve, etc.) (15). Neurogenic dysphagia is caused by damage to one or more nerves innervating the completion of the swallowing maneuver due to direct or indirect causes (16). The results of this study suggest that BAEP testing may be helpful in the diagnosis of neurogenic dysphagia after head injury, and also provide a new diagnostic strategy for screening patients at high risk of dysphagia.

The potential mechanisms underlying the appearance of BAEP alterations in patients with dysphagia are as follows. The five primary neurogenic components of the BAEP originate from distinct loci along the auditory pathway: wave I from the cochlear nerve, wave II from the cochlear nucleus, wave III from the superior olivary complex, wave IV from the lateral lemniscus, and wave V from the inferior colliculus. Given that auditory structures such as the cochlear nucleus and the superior olivary complex are anatomically adjacent to the swallowing-modulatory nuclei located in the medulla oblongata and pons, traumatic lesions involving the brainstem can concurrently compromise both systems. This spatial proximity thus accounts for the co-occurrence of dysphagia and BAEP abnormalities.

Patients with severe brain injury may have damage in the cortical area, brainstem, and peripheral nerves, and some patients may present not only with motor dysfunction, but also with initiation difficulties and cognitive impairment, so they often manifest as multi-site-related dysphagia (17); In addition, traditional subjective assessments, such as the GUSS scale, may be biased, and simple bedside assessments alone cannot meet the complexity of the diagnostic and therapeutic needs of swallowing dysfunction.

In this study, the causes of dysphagia in 106 patients were divided into neurogenic and non-neurological injuries by BAEP detection. There were 60 patients with BAEP-positive and moderate to severe dysphagia assessed by GUSS, which was considered neurogenic dysphagia. Nevertheless, 29 patients exhibited dysphagia despite having negative BAEP results. In addressing this phenomenon, we postulate that the dysphagia in these cases may originate from non-neurological factors, such as endotracheal intubation or tracheostomy, which have been previously identified as significant risk factors for swallowing impairment. Furthermore, in some patients, the dysphagia may result from dysfunction of cortical descending pathways rather than from brainstem damage, thereby preserving normal BAEP transmission. This mechanism also helps to explain why the BAEP positivity rate was highest in the moderate dysphagia group, while it paradoxically declined in the severe dysphagia group. In summary, we consider that BAEP holds diagnostic value for dysphagia of specific etiologies, particularly in patients with brainstem involvement; moreover, a negative BAEP result is also clinically meaningful for the differential diagnosis of the underlying causes of dysphagia.

Because the prognosis of neurogenic dysphagia differs from that of non-neurogenic dysphagia, it is important to determine the cause of dysphagia. At present, post-traumatic dysphagia is only assessed by subjective scales, and most patients cannot cooperate with esophageography, which makes it difficult to diagnose dysphagia early. BAEP is sensitive to detect the presence of nerve damage, responds reliably, and is rarely disturbed by external interference.

This study first stratified patients into three groups—mild, moderate, and severe impairment—based on swallowing function assessment. Unexpectedly, the positive rate of brainstem auditory evoked potentials (BAEP) did not exhibit a linear increasing trend with the severity of dysphagia. Specifically, the moderate dysphagia group showed the highest BAEP positivity, significantly exceeding that of both the mild and severe groups. Notably, in the severe group, 41.7% (5/12) of BAEP recordings were entirely normal. This finding suggests that the pathological mechanisms underlying severe dysphagia do not necessarily involve the brainstem auditory conduction pathways. The non-linear distribution observed—highest BAEP positivity in the moderate group and a normal BAEP in half of the severe group—appears contradictory but may, in fact, arise from selective vulnerability of distinct brainstem functional areas and heterogeneity in the pathways of injury propagation.

In the context of traumatic brain injury (TBI), severe dysphagia can develop via one of two mechanisms: (i) diffuse bilateral damage to the corticobulbar tracts (i.e., pseudobulbar palsy pattern), in which the nucleus ambiguus–nucleus tractus solitarius circuitry within the medulla remains structurally intact but its supranuclear descending drive is interrupted; or (ii) direct destruction of motor nuclei in the ventromedial medulla (e.g., the nucleus ambiguus proper), a region primarily supplied by the paramedian branches of the vertebral artery. In both pathological scenarios that lead to “severe” dysphagia, the lateral lemniscus–inferior colliculus pathway, recorded by BAEP and located in the dorsolateral medulla (supplied by the posterior inferior cerebellar artery, PICA), may remain anatomically preserved, thereby yielding a normal BAEP result.

Based on these findings, we propose that the clinical significance of BAEP in the evaluation of post-TBI dysphagia lies in its etiological typing and anatomical localization. First, when dysphagia co-occurs with a positive BAEP (particularly in the moderate group), it strongly suggests that the injury is located in the dorsolateral pons/medulla; in such patients, rehabilitation interventions targeting the brainstem reticular formation and vestibular–cerebellar pathways (e.g., proprioceptive stimulation, vestibular rehabilitation) should be actively pursued. Second, when dysphagia has reached a severe degree yet BAEP remains normal, clinicians should be highly alerted that the injury predominantly involves the bilateral subcortical descending pathways or the ventral medullary motor nuclei; for these patients, rehabilitation strategies should preferentially focus on peripheral efferent pathways, such as vagus nerve electrical stimulation or cricopharyngeal balloon dilatation.

Further, using the moderate-to-severe dysphagia graded by the GUSS as the gold standard and BAEP positivity as the test indicator, we constructed a receiver operating characteristic (ROC) curve. The analysis yielded a sensitivity of 86.96% (60/69), a specificity of 54.05% (20/37), and an area under the ROC curve (AUC) of 0.705. These results indicate that BAEP possesses moderate diagnostic accuracy for dysphagia. In the early phase of TBI, when patients are unconscious and cannot cooperate with bedside swallowing assessments, BAEP can be performed at the bedside in an objective and quantitative manner, providing an early warning signal regarding whether brainstem function has been compromised.

In summary, BAEP demonstrates potential clinical utility in patients with post-TBI dysphagia, serving both as an early warning indicator and as a tool for etiological subtyping.

## Limitation

The follow-up time of this study was short, and the long-term recovery of swallowing function was not followed up in the long term. Another limitation is that bedside audiogram testing poses considerable difficulties due to the suboptimal level of consciousness or limited cooperation capacity in some patients. Moreover, audiogram testing demands stringent environmental quietness, which is challenging to achieve in the neurosurgical intensive care unit. Furthermore, patients with craniocerebral injury complicated with dysphagia in only one general hospital were selected as the study population, which was a single-center study, and there may be bias in sample inclusion, which may have a certain impact on the results of this study.

## Data Availability

The raw data supporting the conclusions of this article will be made available by the authors, without undue reservation.
